# Clinical responses following inspiratory muscle training in exercise-induced laryngeal obstruction

**DOI:** 10.1007/s00405-021-07214-5

**Published:** 2021-12-26

**Authors:** Astrid Sandnes, Tiina Andersen, Hege Havstad Clemm, Magnus Hilland, John-Helge Heimdal, Thomas Halvorsen, Ola Drange Røksund, Maria Vollsæter

**Affiliations:** 1 Department of Internal Medicine, Innlandet Hospital Trust, Gjøvik, Norway; 2 Department of Physiotherapy, Haukeland University Hospital, Bergen, Norway; 3 Department of Pediatrics, Haukeland University Hospital, Bergen, Norway; 4 Department of Otolaryngology/Head and Neck Surgery, Haukeland University Hospital, Bergen, Norway; 5 Department of Surgery, Haukeland University Hospital, Bergen, Norway; 6 Institute of Clinical Medicine, University of Bergen, Bergen, Norway; 7 Institute of Surgical Science, University of Bergen, Bergen, Norway; 8 Thoracic Department, Norwegian Advisory Unit on Home Mechanical Ventilation, Bergen, Norway; 9 Department of Sports Medicine, Norwegian School of Sport Sciences, Oslo, Norway; 10 The Faculty of Health and Social Sciences, Western Norway University of Applied Sciences, Bergen, Norway

**Keywords:** Laryngeal obstruction, Exercise, Dyspnea, Stridor, Inspiratory muscle training

## Abstract

**Purpose:**

Exercise-induced laryngeal obstruction (EILO) is relatively common in young people. Treatment rests on poor evidence; however, inspiratory muscle training (IMT) has been proposed a promising strategy. We aimed to assess laryngeal outcomes shortly after IMT, and to compare self-reported symptoms with a control group 4–6 years later.

**Methods:**

Two groups were retrospectively identified from the *EILO-register* at Haukeland University Hospital, Norway; one group had received only information and breathing advice (IBA), and another additionally IMT (IBA + IMT). At diagnosis, all participants performed continuous laryngoscopy during exercise (CLE), with findings split by glottic and supraglottic scores, and completed a questionnaire mapping exercise-related symptoms. After 2–4 weeks, the IBA + IMT-group was re-evaluated with CLE-test. After 4–6 years, both groups were re-assessed with a questionnaire.

**Results:**

We identified 116 eligible patients from the *EILO-register*. Response rates after 4–6 years were 23/58 (40%) and 32/58 (55%) in the IBA and IBA + IMT-group, respectively. At diagnosis, both groups rated symptoms similarly, but laryngeal scores were higher in the IBA + IMT-group (*P* = 0.003). After 2–4 weeks, 23/32 in the IBA + IMT-group reported symptom improvements, associated with a decrease of mainly glottic scores (1.7–0.3; *P* < 0.001), contrasting unchanged scores in the 9/32 without symptom improvements. After 4–6 years, exercise-related symptoms and activity levels had decreased to similar levels in both groups, with no added benefit from IMT; however, full symptom resolution was reported by only 8/55 participants.

**Conclusion:**

Self-reported EILO symptoms had improved after 4–6 years, irrespective of initial treatment. Full symptom resolution was rare, suggesting individual follow-up should be offered.

**Supplementary Information:**

The online version contains supplementary material available at 10.1007/s00405-021-07214-5.

## Introduction

Exercise-induced laryngeal obstruction (EILO) is an umbrella term describing inappropriate and transient adduction of laryngeal structures during exercise causing breathlessness and/or stridor [1], and is reported to affect 5–7% of young people [2, 3]. Symptoms typically peak at maximal exercise or immediately after, with respiratory distress, prolonged inspiration, a chocking sensation, and sometimes panic [4]. Symptoms of EILO are sometimes interpreted as exercise-induced asthma (EIA) [1, 5], a situation likely leading to mismanagement of both conditions [6]. Besides limiting otherwise healthy adolescents in their physical activities [6, 7], undiagnosed respiratory problems might reduce quality of life and disrupt participation on sports arenas [8, 9]. Individuals with EILO represent a diverse group and range from sedentary youngsters who might be affected in light activities, to top athletes whose performance is affected only when ventilation requirements are very high [10].

EILO is diagnosed by continuously visualizing the larynx with a flexible laryngoscope during maximal exercise (continuously laryngoscopy exercise test, CLE-test) [11]. Laryngeal obstruction can occur at the supraglottic or glottic level or involve both levels, and one may differentiate between EILO subgroups with mainly glottic or supraglottic obstruction, possibly associated with different causal mechanisms [12]. Laryngeal findings can be graded according to severity, using, e.g., CLE-scores, where higher scores indicate more obstruction [12, 13]. However, the perception of breathlessness is subjective [14, 15], and associations with CLE-scores are poorly described [16]. Treatment of EILO is largely based on empirical data [4, 12]. Most conservative strategies focus on making patients properly aware of their breathing pattern, structured breathing advice, and various breathing practices [1, 17–19]. Tailoring treatment modalities based on EILO subgroups or CLE-scores is still based on weak evidence, except for severe supraglottic EILO where supraglottoplasty has been reported successful [20].

Inspiratory muscle training (IMT) has been proposed a promising tool to treat EILO. Preliminary research suggest that IMT primarily improve the glottic component; however, randomized controlled studies are required to confirm these reports [22, 24-28]. IMT is based on breathing exercises performed with a resistance applied during inspiration, focusing on enhanced coordination and strengthening of the inspiratory muscles [21]. The effect might rest on the phasic relationship that exist between the diaphragm and the main laryngeal abductor (the posterior cricoarytenoid muscle); the former contracting immediately before the latter [23].

We aimed to assess improvements of self-reported symptoms and laryngeal outcomes in EILO shortly after treatment with standardized information and breathing advice (IBA) plus 6 weeks of IMT. After 4–6 years, we conducted a questionnaire-based follow-up in the same individuals, comparing their self-reported symptoms with a control group with EILO who had only received IBA.

## Methods

### The EILO-register and CLE-test

**Fig. 1:**
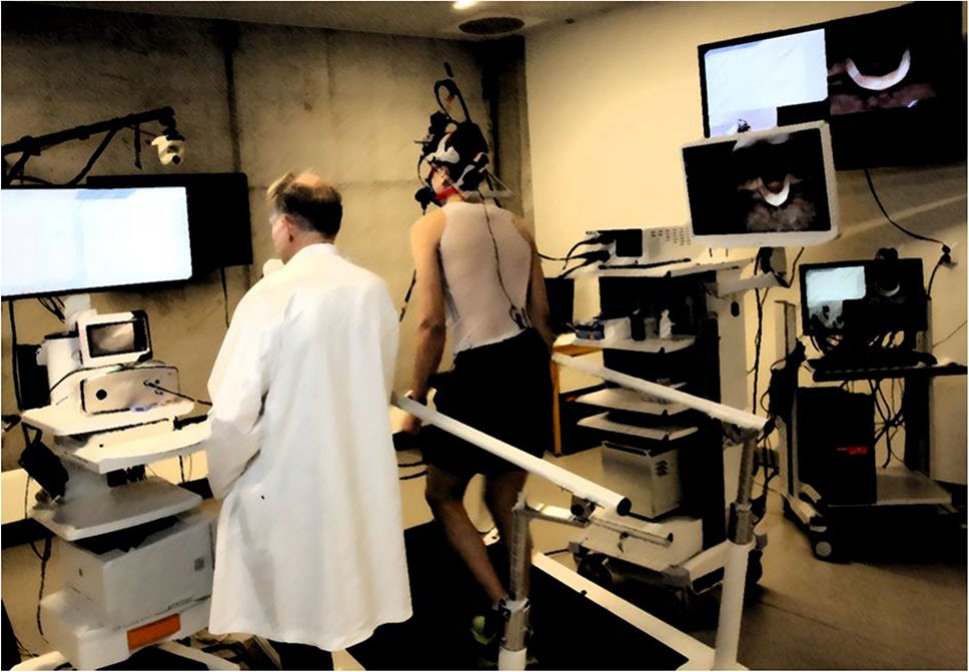
The continuous laryngoscopy exercise (CLE) test**.** An integrated setup with a trans-nasal flexible fiberoptic laryngoscope (Olympus ENF-P3^®^, Tokyo, Japan), diameter 3.5 mm, introduced after applying a decongestive nasal spray (Rhinox^®^) and local anesthesia (Xylocaine^®^), and secured in a position allowing for a good view of the laryngeal entrance, including both supraglottic structures and the vocal folds. Continuous video recordings from the laryngoscope, a film of the upper part of the body, and breath sounds are obtained simultaneously throughout a maximal cardiopulmonary exercise test, and stored in one single file for later evaluation. The method is widely used and applied as previously described [11]

**Fig. 2:**
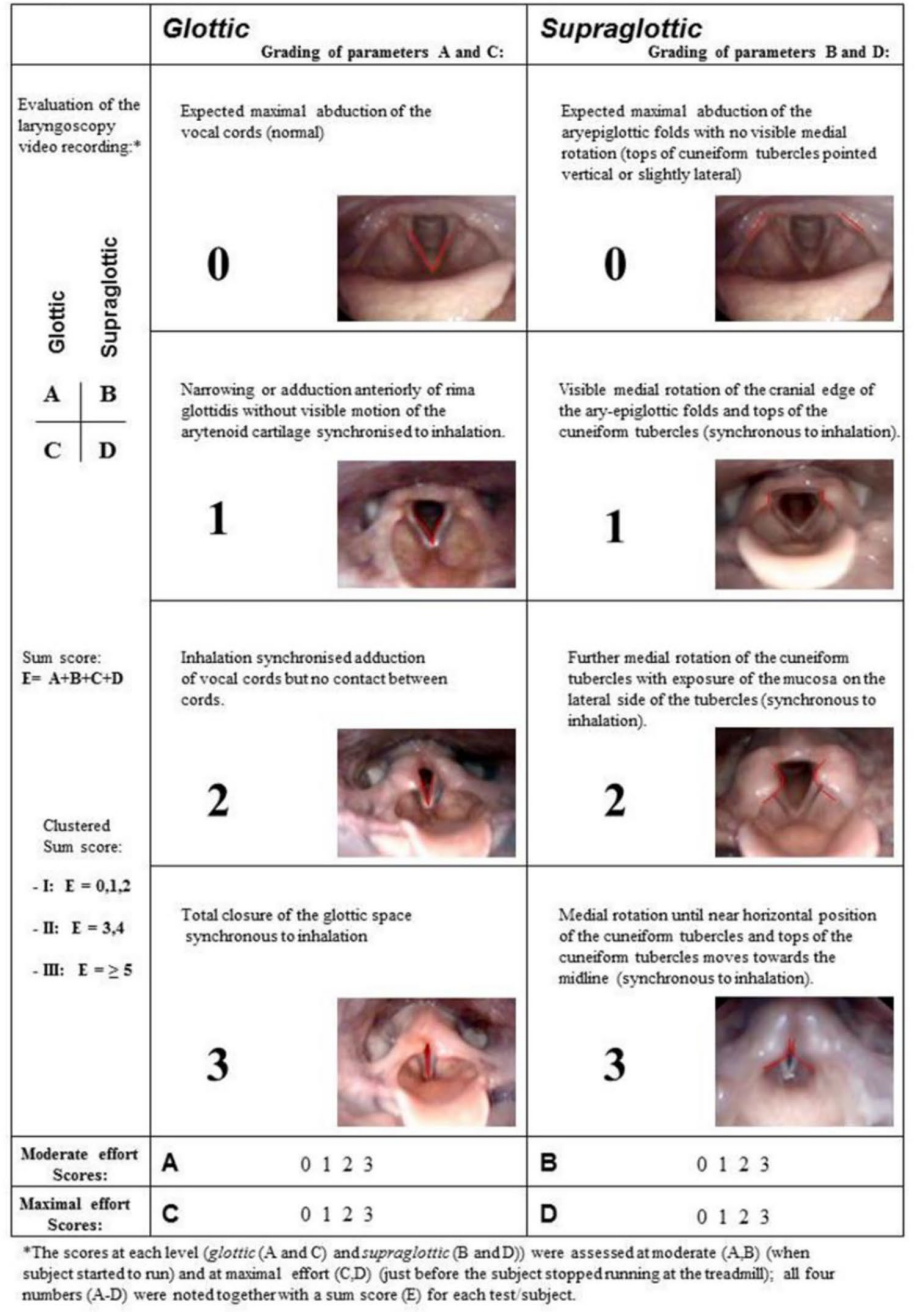
Grading system of laryngeal obstruction according to Maat et al. [13]. Reprinted by permission from Springer Nature. European Archives of Oto-Rhino-Laryngology, Copyright © 2009, Dec;266(12):1929–36. Epub 2009 Jul 8 (https://doi.org/10.1007/s00405-009-1030-8)

Since 2013, more than 99 percent of patients diagnosed with EILO at our institution have been consecutively enrolled in the *Bergen EILO-register*, where background demographics and self-reported questionnaires are stored together with data from the CLE-test, which contains laryngoscopy findings from rest to peak exercise, a soundtrack, and a film of the upper part of the body. CLE-tests are routinely performed as described previously [11], for details please see Fig. 1. The CLE-test permits grading of laryngeal obstruction from 0 (complete patency) to 3 (almost complete closure) at the glottic and supraglottic level (the CLE-score, see Fig. 2) [13]. The CLE recordings were rated in retrospect and in random order by two experienced reviewers who were both blinded to the identity of the person and the clinical situation (i.e., if the film was obtained at diagnosis or 2–4 weeks after IBA + IMT). The findings were split by supraglottic and glottic scores, and disagreements were solved by consensus.

### Participants and study design

**Fig. 3:**
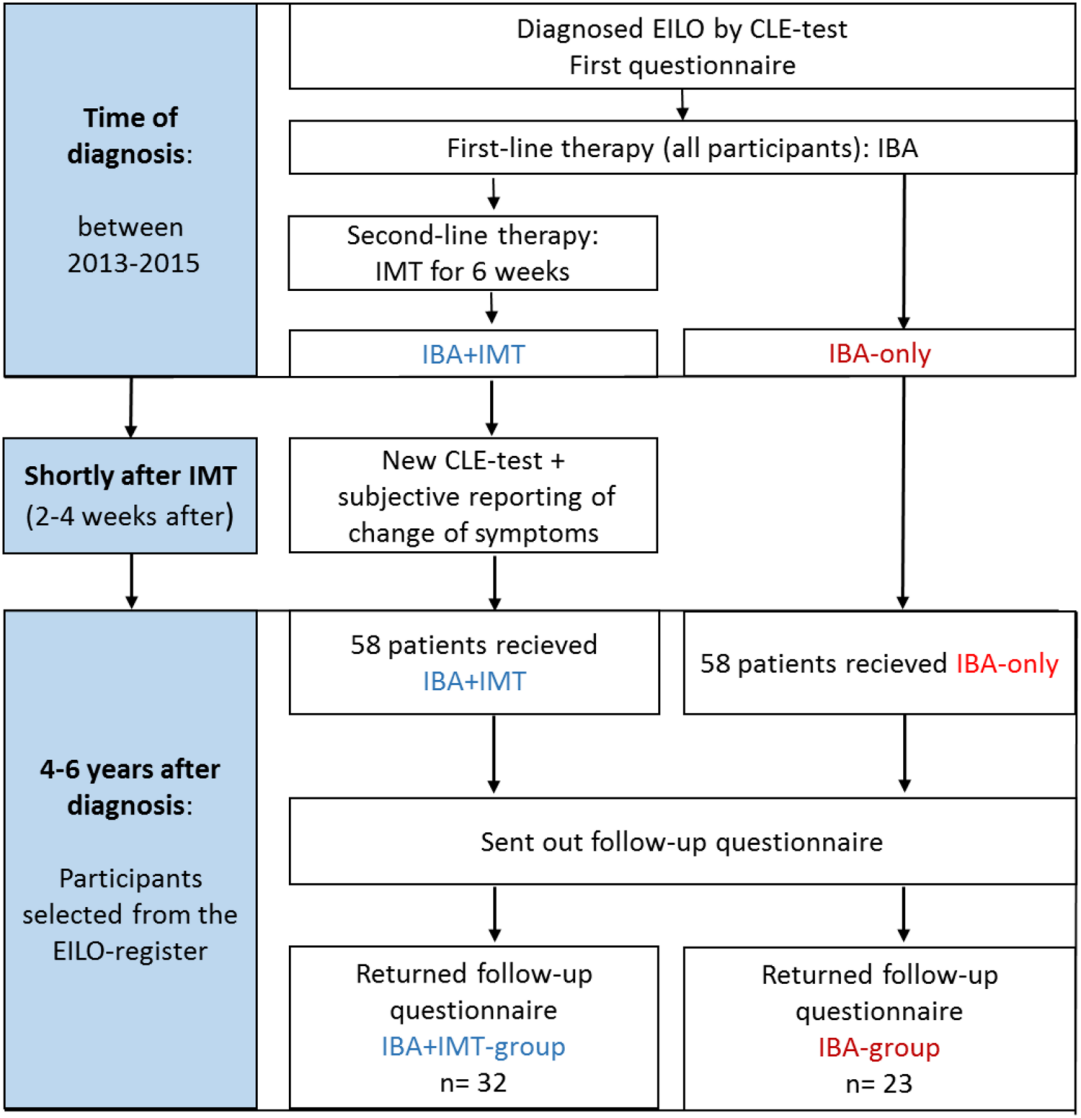
Overview of study design and participants included in the study of subjects diagnosed with exercise-induced laryngeal obstruction (EILO) at the outpatient clinic at Haukeland University Hospital in Bergen, Norway between 2013 and 2015. All participants received information and physician guided breathing advice (IBA), and one group additionally received inspiratory muscle training (IMT) at diagnosis

The participants were retrospectively identified from the *Bergen EILO-register*, based on access to a diagnostic CLE-test performed during 2013–2015, followed by treatment with standardized IBA plus 6-weeks of IMT, and a subsequent re-evaluation with CLE after 2–4 weeks (IBA + IMT-group). Only patients with a documented compliance to the IMT training exceeding 70%, assured by the memory card of the device, were included. A control group was identified, based on a diagnostic CLE-test and treatment with only IBA during 2013–2014 (IBA-group). A questionnaire mapping self-reported symptoms were completed by all participants at diagnosis and mailed to both groups at a follow-up 4–6 years later (Fig. 3).

The study was approved by the Committee on Medical Research Ethics of Western Norway (REK number 2016/1898). Informed written consent was obtained from participants and/or guardians.

### Information and breathing advice (IBA) with biofeedback

Standardized IBA is provided by physicians as first line treatment to all patients diagnosed with EILO at our institution. IBA consists of information about the diagnosis, its benign nature and structured breathing advice while observing the laryngeal responses on the monitor (biofeedback). The breathing advice entails guidance in posture, relaxation of the shoulder girdle, diaphragmatic breathing, avoidance of noisy breathing/stridor during exercise, early recognition of initial signs of breathing problems, and regaining control when such signs arise. Patients are encouraged to maintain their level of physical activity, and to practice breathing advice while exercising.

After the IBA session, patients were (and still are) assigned to second-line treatment depending on the severity of the laryngeal obstruction and the motivation of the patient. At the time the participants were diagnosed, second-line treatment consisted of either speech therapy or inspiratory muscle training (IMT) for 6 weeks. As no hard data existed to prioritize between these two modalities, they were used interchangeably, based on availability. Only those enrolled in IMT treatment were relevant to this particular follow-up study.

### Inspiratory muscle training (IMT)

Patients found eligible for IMT were recruited at time of diagnosis, after the IBA had been provided. IMT instructions were then given, and standardized training was performed over the following 6 weeks, using a resistive loading device, Respifit S^®^ (*Biegler GmbH, Mauerbach, Austria*), for details please see Online resource 1, or a previous communication [27]. The protocol alternates between two modes of resistance; A) inspiratory resistance ≥ 80% of maximum produced mouth pressures (Pi_max_) [29], and B) inspiratory resistance: 60–80% of Pi_max_. Evaluation of IMT was performed 2–4 weeks after the 6-week training period with a second CLE-test.

### Questionnaire

The participants (and/or guardians) completed a questionnaire at diagnosis, then 2–4 weeks after IMT (only the IBA + IMT-group), and at follow-up 4–6 years after diagnosis (all participants). The questionnaire (Online resource 2) covered medical history, development (Q–A.1–8) and significance (Q-B.1–2) of the breathing problem, symptom perception (Q1–18) and level of physical activity. A reminder was sent after 2 months to those who did not respond.

### Study outcomes and statistical methods

This was a register-based descriptive follow-up study with self-reported symptoms as primary outcome, supplemented with CLE-scores obtained by a new CLE-test 2–4 weeks after treatment in the IBA + IMT-group. A secondary outcome was self-reported level of physical activity. CLE-scores are by nature categorical [13], but are reported as means with 95% confidence intervals (CI) which is considered to provide more information than medians and interquartile ranges when there are few categories [30]. Descriptive data was presented with counts and percentages. After checking for normality, the symptom scores (at diagnosis and follow-up) were compared with t-tests and one-way ANOVA, as appropriate. Mann–Whitney *U* test was applied for non-normally distributed data. Analyses were performed with SPSS version 24 (SPSS, Chicago, IL, USA). A *P* value < 0.05 was considered significant.

## Results

### Participants

Fifty-eight participants from the *EILO-register* were eligible for inclusion in the IBA + IMT-group, of whom 32/58 (55%) participants returned the questionnaire 4–6 years after diagnosis. Correspondingly, 58 participants, consecutively treated only with IBA, were approached as controls, of whom 23/58 (40%) participants returned the questionnaire (Fig. 3). The two groups had similar ratings of self-reported symptoms at diagnosis. Two participants in the IBA-group had to be excluded, as they had later been treated with supraglottoplasty. Demographics and symptoms at diagnosis did not differ between responders and non-responders (Online resource 3), except that non-responders in the IBA + IMT-group reported less problems when physically active (Q12). Mean (range) follow-up time was 59 (46–73) and 60 (54–71) months in the IBA and IBA + IMT-group, respectively. Demographic data are listed in Table 1. Although symptom scores were similar at diagnosis, CLE-scores were higher in the IBA + IMT-group (*P* = 0.003), which was as expected in a group selected for second-line therapy. Thirteen of the 32 IBA + IMT-group responders took part in a previously published study on IMT [27]. None reported any side effects at follow-up after training with IMT.

**Table 1:** Demographic data obtained at diagnosis from subjects identified with exercise induced laryngeal obstruction (EILO) at the outpatient clinic at Haukeland University Hospital in Norway

	Participants^a^		Group comparisons at diagnosis
	IBA + IMT	IBA	*P* value
Number (percent females)	32 (90)	23 (56)	
BMI at diagnosis, mean (95% CI)	21 (20–22)	21 (20–22)	0.993
Age symptom debut, mean (range)	12.6 (1.5–20)	10.5 (5–15)	0.052
Age at diagnosis, mean (range)	17.5 (10–30)	15.2 (12–21)	0.019
Age at follow-up, mean (range)	22.7 (15–36)	20.4 (17–26)	0.036
FEV1, % of predicted (95% CI)	112% (108–116)	110% (106–114)	0.936
EIA diagnoses at first visit; yes/no/unsure (*n*)	3/17/12	3/16/4	
Asthma medication before referral; yes/no (*n*)	6/15	8/21	
Activity hours, median per week, at diagnosis vs. follow-up	≥ 7 vs. 4–6*	≥ 7 vs. 4–6*	0.667
Level of sports activity at diagnosis vs. at follow-up (*n*)			0.803
No organized activity	2 vs. 19*	3 vs. 13*	
Competing at local/regional level	16 vs. 7*	16 vs. 6*	
Competing at national level	10 vs. 4*	3 vs. 3	
Competing at international level	4 vs. 2	0 vs. 0	

All subjects had received information and breathing advice (IBA) and one group had additionally received inspiratory muscle training (IBA + IMT group). Data obtained at diagnosis and at follow-up after 4–6 years

*IMT* inspiratory muscle training, *IBA* information and breathing advice, *CI* confidence interval

*P* values were calculated using Students t-tests or Mann–Whitney-*U*-test as appropriate

*Indicates a significant change in activity level from diagnosis to follow-up after 4–6 years

^a^Items where numbers do not add up to the total group number are due to missing answers on questionnaire

### Re-evaluation, 2–4 weeks after IMT (IBA + IMT-group only)

**Fig. 4:**
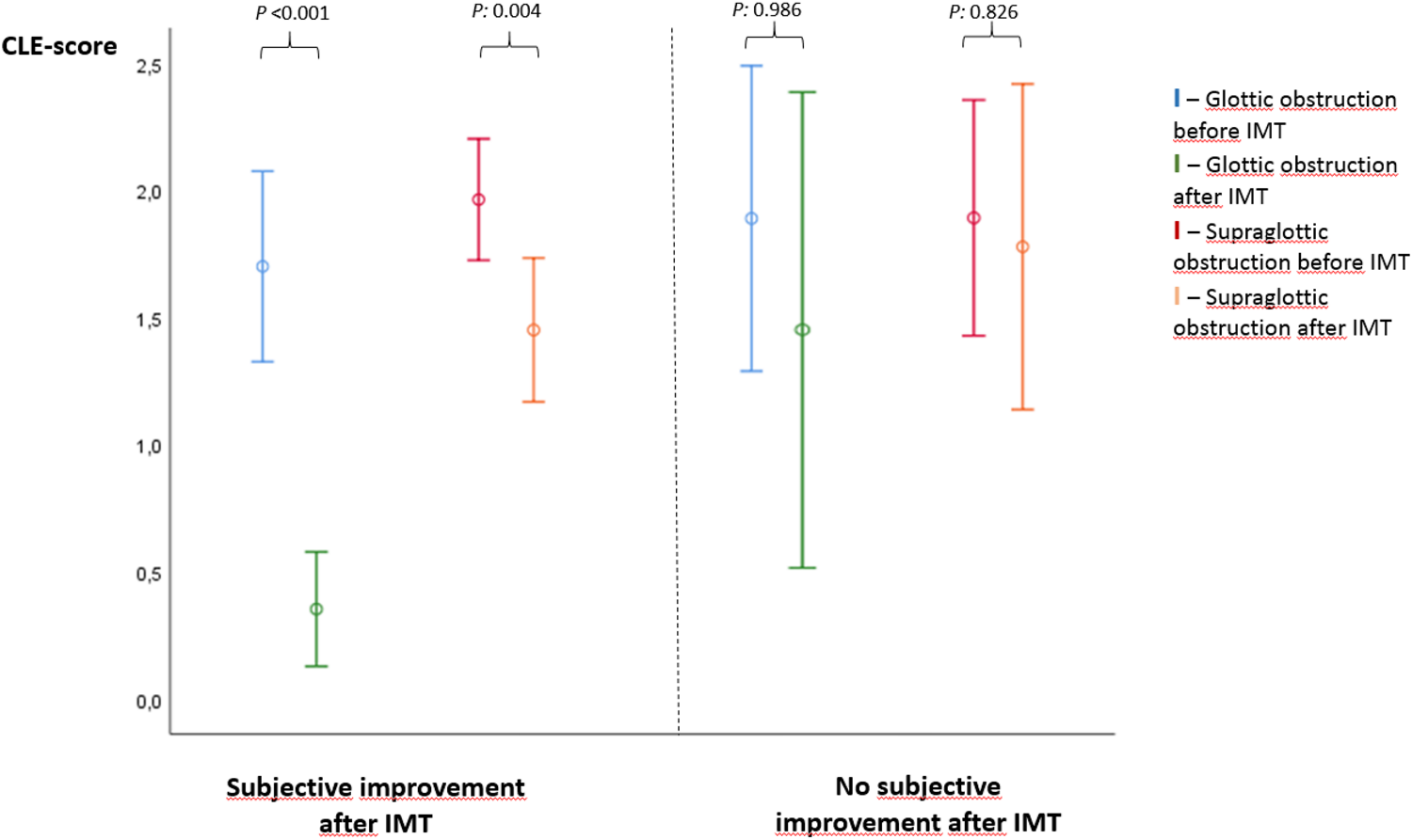
Laryngeal obstruction (evaluated by CLE-scores at glottic and supraglottic level) in subjects diagnosed with exercise induced laryngeal obstruction (EILO) at Haukeland University Hospital and treated with information and breathing advice (IBA) and additionally received 6 weeks of inspiratory muscle training (IBA + IMT group). The group is split according to reports of subjective improvement 2–4 weeks after IMT. *P* values refer to mean change of glottic and supraglottic CLE-scores after 6 weeks of IMT

Symptom scores had improved in most participants (23/32; 72%), and improvements were associated with reduced CLE-scores, particularly at the glottic level (*P* < 0.001). In participants without subjective improvement (9/32; 18%), the CLE-scores were unchanged (*P*.565) (Table 2, Fig. 4).

**Table 2:** Laryngeal obstruction (evaluated by CLE-scores) in subjects diagnosed with exercise induced laryngeal obstruction (EILO) at Haukeland University Hospital

	IBA + IMT			IBA	
	Subjective improvement	No subjective improvement	*P* value^b^	*n* = 23	*P* value^c^
	2–4 weeks after IMT (*n* = 23)	2–4 weeks after IMT (*n* = 9)			
CLE-scores (total) at diagnosis	3.7 (3.2–4.1)	3.9 (3.1–4.7)	0.565	2.7 (2.3–3.2)	0.003
Glottic score	1.7 (1.3–2.0)	1.9 (1.4–2.6)	0.444	1.2 (0.9–1.6)	0.029
Supraglottic score	2.0 (1.8–2.3)	2.0 (1.4–2.6)	1	1.6 (1.3–2.0)	0.075
CLE-scores (total) shortly after IMT^a^	1.8 (1.4–2.2)	3.4 (2.2–4.6)	< 0.001	Not done	
Glottic score	0.3 (0.1–0.6)	1.6 (0.6–2.6)	< 0.001	Not done	
Supraglottic score	1.5 (1.2–1.8)	1.8 (1.0–2.5)	0.346	Not done	
The level of laryngeal obstruction, *n*					
Supraglottic > glottic	9	3		11	
Supraglottic < glottic	3	2		6	
Supraglottic = glottic	11	4		6	

All subjects had received information and breathing advice (IBA) and one group had additionally received inspiratory muscle training (IBA + IMT group). The IBA + IMT group is split according to reports of subjective improvement 2–4 weeks after IMT

Figures are numbers (*n*) or means (95% confidence intervals)

*IMT* inspiratory muscle training, *IBA* information and breathing advice, *CI* confidence interval, *CLE-score* continuous laryngoscopy exercise score, grading of laryngeal obstruction according to Maat et al. [1],

^a^All subjects in the IBA + IMT group completed a new CLE-test 2–4 weeks after six weeks of IMT for evaluation

^b^Comparison of scores with Student’s *t* test between subjects with subjective improvement shortly after IMT and no subjective improvement shortly after IMT

^c^Comparison of scores with Student’s *t* test between IBA + IMT group (combined subjective improvement shortly after IMT and no subjective improvement, not tabulated) and the IBA-group

### Follow-up 4–6 years after diagnosis (all participants):

#### The significance of the breathing problem and the severity of the symptoms

Most questionnaire items describing symptom severity had improved, with no differences between the two treatment groups (Table 3). There were no associations between a positive response at the re-evaluation 2–4 weeks after IBA + IMT treatment and symptom descriptions at the 4–6-year follow-up. Only five (16%) in the IBA + IMT-group and three (13%) in the IBA-group stated, “I no longer have a breathing problem” (Q–A.8). Seven (22%) in the IBA + IMT-group and 9 (39%) in the IBA-group stated, “the problem is unchanged and bothers me as much as before” (Q–A.2). One in the IBA + IMT-group reported worsening of the breathing problem (Q–A.1) (Table 4). In both groups, the impact of the breathing problem was reduced when “considering life overall” (Q-B.2), and the number responding positively to the question “I can control my symptoms when I get them” (Q18) was numerically increased; although significant only in the IBA + IMT-group (*P* 0.018) (Table 3).

**Table 3:** Self-reported data obtained at diagnosis and at follow-up 4–6 years after being diagnosed with exercise induced laryngeal obstruction (EILO) at Haukeland University Hospital

Questions asked on exercise related issues at follow-up 4–6 years after diagnosis	IBA + IMT			IBA			*P* value^b^
	*n* = 32		*P* value^a^	*n* = 23		*P* value^a^	
	At diagnosis	At follow-up		At diagnosis	At follow-up		
Q1. I have trouble breathing in	4.2 (3.8–4.6)	2.5 (2.1–3.0)	< 0.001	4.3 (3.9–4.8)	2.3 (1.8–2.7)	< 0.001	0.46
Q3. I feel tightness/pain in my throat	3.6 (3.1–4.2)	2.3 (1.7–2.8)	0.002	3.9 (3.3–4.6)	2.1 (1.6–2.7)	< 0.001	0.486
Q7. I feel like I'm being choked	3.7 (3.1–4.2)	2.0 (1.5–2.5)	< 0.001	3.3 (2.6–4.1)	1.8 (1.3–2.3)	< 0.001	0.528
Q8. I become dizzy, nauseous and feel like I'm going to faint	2.8 (2.3–3.4)	1.6 (1.2–1.9)	< 0.001	2.4 (1.7–3.2)	1.5 (1.0–2.0)	0.008	0.11
Q9. The symptoms come on fast	3.8 (3.3–4.3)	2.4 (1.9–2.8)	< 0.001	3.9 (3.4–4.4)	1.9 (1.4–2.5)	< 0.001	0.167
Q11. I feel panic	2.9 (2.2–3.6)	1.3 (1.1–1.6)	< 0.001	2.6 (1.8–3.4)	1.7 (1.1–2.3)	0.009	0.268
Q12. I have problems breathing when I am physically active	4.0 (3.5–4.5)	2.9 (2.3–3.4)	0.002	4.1 (3.6–4.7)	2.6 (2.0–3.1)	< 0.001	0.201
Q13. I can hear unusual or wheezing sounds when I breathe	3.9 (3.4–4.5)	2.3 (1.8–2.7)	< 0.001	3.2 (2.3–4.1)	1.8 (1.4–2.3)	0.001	0.754
Q14. My symptoms prevent me from training/exercising	3.0 (2.4–3.6)	1.6 (1.3–2.0)	< 0.001	2.7 (2.1–3.3)	1.6 (1.0–2.2)	< 0.001	0.119
Q15. I become afraid when I get symptoms	2.8 (2.1–3.6)	1.2 (1.0–1.4)	< 0.001	2.6 (1.8–3.3)	1.6 (1.0–2.2)	0.002	0.171
Q16. My symptoms prevent me pushing myself when exercising	3.2 (2.6–3.8)	2.0 (1.6–2.4)	< 0.001	3.1 (2.4–3.8)	1.7 (1.0–2.3)	0.002	0.174
Q18. I can control my symptoms when I get them	2.6 (2.0–3.2)	3.3 (2.9–3.8)	0.018	2.3 (1.7–2.8)	2.8 (2.1–3.6)	0.064	0.299
Q-B.2: How much do your breathing problems effect you NOW?	2.9 (2.6–3.2)	2.0 (1.7–2.3)	< 0.001	2.9 (2.4–3.5)	1.8 (1.3–2.3)	< 0.001	0.436
Q-B.1: How much did your breathing problems effect you before?	–	3.3 (3.1–3.6)	0.016^c^	–	3.1 (2.6–3.5)	0.426^c^	

All participants had received information and breathing advice (IBA) and one group had additionally received inspiratory muscle training (IBA + IMT group)

Figures are means (95% confidence intervals)

Answers to Q1–Q18 were based on ordinal scale from 1 to 5: 1: never, 2: occasionally, 3: often, 4: nearly always, 5: always

Answers to Q-B1-2 were based on scale from 1 to 5: 1: not at all, 2: a little, 3: quite a lot, 4: a great amount, 5: crippling

^a^Compares means in each group at diagnosis versus at follow-up with student’s paired *t* test

^b^Compares means at follow-up to each question between the IBA + IMT group and the IBA-group with student *t* test

^c^Compares mean answer at follow-up to question Q-B1: “how much did your breathing problems effect you before?” (by retrospective recall) and at time of diagnosis to QB-2: “how much do your breathing problems effect you?” with student’s paired *t* test, mean difference was in IBA + IMT group: − 0.40 and in IBA group − 0.17

**Table 4:** Self-reported data obtained at follow-up 4–6 years after being diagnosed with exercise induced laryngeal obstruction (EILO) at Haukeland University Hospital

Questions asked at follow-up 4–6 years after diagnosis The IBA + IMT group split according to responses at assessment 2–4 weeks after IMT	IBA + IMT		IBA	*P* value^a^
	Subjective improvement 2–4 weeks after IMT (*n* = 23)	No-subjective improvement 2–4 weeks after IMT (*n* = 9)	*n* = 23	
Reported as numbers yes/no or unsure	Yes/no or unsure	Yes/no or unsure	Yes/no or unsure	
“Since time of diagnosis”				
Q-A.1:“The breathing problem have got worse”	0/23	1/8	0/23	0.240
Q-A.2: “The breathing problem is unchanged”	6/17	0/7	9/14	0.296
Q-A.4: “I have less breathing problems because I am less active”	11/12	2/7	10/13	0.884
Q-A.7: “The breathing problem has improved”	13/10	7/2	9/14	0.183
Q-A.8: “I no longer have a breathing problem”	3/20	2/7	3/2 0	0.121

All participants had received information and breathing advice (IBA) and one group had additionally received inspiratory muscle training (IBA + IMT group). The IBA + IMT group is split according to reports of subjective improvement 2–4 weeks after IMT

*IBA* information and physician guided breathing advice), *IBA + IMT* received IBA and additionally received inspiratory muscle training (IMT) for 6 weeks

^a^Comparison of scores with Mann-*U*-Whitney test between IBA + IMT group (combined subjective improvement shortly after IMT and no subjective improvement, not tabulated) and the IBA-group

#### Physical activity level at follow-up

Twenty-three (73%) in the IBA + IMT-group and 12 (52%) in the IBA-group reported reduced level of physical activity (*P* 0.803). Thirteen (41%) in the IBA + IMT-group and 10 (44%) in the IBA-group reported “less breathing problems because I am less active” (Q–A.4) (Table 4). Eight (25%) in the IBA + IMT-group and five (22%) in the IBA-group still reported “nearly always or always” to the question “I have breathing problems when I am physically active” (Q12). However, most participants in both the IBA + IMT (87%) and the IBA (65%) group answered “never” to the question “Symptoms prevent me from exercising” (Q14) (Table 3).

### Individual differences at follow-up

There were extremes as regards self-reported symptoms at follow-up in both groups and in both directions; some reported to be symptom-free, and some reported still being severely affected. For example, in both treatment groups, responses at follow-up to the question “How much do your breathing problems effect you?” could vary from “not at all or a little” to “crippling” (Q-B.2*)*. None in the IBA + IMT-group and two in the IBA-group reported panic (Q11) or to be afraid when symptoms arise (Q15).

## Discussion

This is the first follow-up study aiming to evaluate self-reported symptoms and CLE-scores after IMT used to treat EILO. We found that self-reported symptoms and laryngeal findings had improved in most participants shortly after treatment with standardized IBA plus 6 weeks of IMT. Symptom improvement was associated with improved CLE-score, particularly at the glottic level. After 4–6 years, self-reported symptoms had improved to similar levels both in the IBA + IMT-group and in the IBA-group, irrespective of laryngeal findings and symptom reports shortly after the IMT. Symptom resolution was rare, and respiratory problems still disturbed most participants during exercise. The level of physical activity had decreased in most participants during the 4–6-year follow-up.

### Strengths and limitations of the study

The major strengths of the study were enrolment of participants who were considered typical of patients seeking treatment for EILO, diagnostic accuracy ensured by a CLE-test performed in all participants at inclusion [1], and that a control group treated with IBA only were included for comparison at the 4–6-year follow-up. Recruitment was based on retrospective identification of all eligible participants from a nationwide EILO-register with high cover ratio. Major weaknesses were that information at the 4–6-year follow-up was based only on self-reports, the retrospective nature of the study and therefore a risk of recall bias, no randomization of participants between the two treatment groups, a high attrition rate, and relatively few participants. The lack of a control group at the time when the short-term influence of IMT was evaluated, prevents us from reliably ascribing the early changes in participants using IMT to the IMT itself—since IBA had also been provided. The questionnaire was not a validated instrument in relation to EILO, and relationships between answers therefore challenging to elucidate in some instances. However, key items were selected from sources validated in EILO or other respiratory contexts [31, 32]. Diagnostic characteristics from the time of the diagnosis were similar between responders and non-responders; however, we had no way of establishing their treatment motivation. The questionnaire required the participants to report utilization of other treatment modalities (for example psychological counselling) during the years that had passed since they had been diagnosed. Our response rate was in line with some [33] and lower than other studies [34–36], but the study still represents one of the few studies evaluating symptoms from EILO over time.

### The challenges of self-reported symptoms and their relation to objective findings

Dyspnea is a complex and highly subjective feature [15], consisting of both a sensation of breathlessness and an emotional interpretation [37], i.e., the individual’s coping mechanisms to handle the breathlessness. As dyspnea can only be perceived by the individual who experience it, assessment must rest on self-report [38]. The questionnaire used in this study, addressed domains of sensory-perceptual experience and symptom impact or burden, as recommended when assessing dyspnea [38]. We know from other studies that self-reported instruments tend to overestimate intensity and duration of physical activity [39], and that poor physical condition often explain self-reported exercise-induced dyspnea [40], factors we could not control for in our study. Bias by recall or in terms of altered positive and negative expectations related to the experience of symptoms over time, may also be of importance. This mechanism may have been involved in the IBA + IMT-group, where the significance of the breathing problem was scored lower when asked at diagnosis, compared to when retrospectively confronted with the same question at follow-up (Table 3).

The study highlights numerous challenges relating to subjective symptom-reporting versus objective findings in general [41]. Specifically, the study underlines that individuals’ perception of dyspnea do not necessarily correspond to their CLE-scores. Symptom scores at diagnosis were similar in both groups, despite more advanced laryngeal obstruction (higher CLE-scores) in the IBA + IMT-group. Similar levels of self-reported symptoms at diagnosis facilitated a direct comparison at the 4–6 years follow-up, when symptom descriptions were still similar between the two groups, although at lower levels. Interestingly, at the CLE re-evaluation shortly after the IMT, subjective improvements of self-reported symptoms were associated with improved CLE-scores. However, this initial short-term response was seemingly unrelated to symptom scores 4–6 years later. A better understanding of the diagnostic implications and interactions between laryngeal obstruction and individuals’ perception of dyspnea is clearly an unmet need in this field of respiratory medicine [5, 16].

### The age and gender distribution of EILO in this study and in general

Participants were recruited from the *Bergen EILO-register*, which has a high cover ratio, suggesting they were representative for typical EILO patients referred for second-line care. This notion is supported by an age distribution in line with most clinical EILO studies [2]. The participants of the IBA + IMT-group was slightly older than in the IBA-group, perhaps reflecting that older patients felt more motivated for second-line therapy. The female predominance was even higher than reported by others [2, 12], especially in the IBA + IMT-group. Although poorly investigated, gender-dependent anatomical changes of the larynx during puberty suggest a smaller inner diameter of the larynx in females that may explain a female predominance in young adults with EILO [42]. Females also tend to report higher symptom scores in health surveys [39], a factor that might contribute to more females considered for second-line therapy for EILO.

### Development of EILO and physical activity

At follow-up 4–6 years after diagnosis, most participants had reduced their level of physical activity, and about half the participants reported that this was the cause of experiencing less breathing problems. The typical age for the debut of EILO corresponds to a period when physical activity often starts to decline [43, 44]. We do not know the natural course of EILO, but the few studies investigating this issue suggest that EILO persists over time [34–36]. This is consistent with our findings, with only a few participants reporting full resolution of their breathing problems. A study that re-examined individuals with EILO after 20 years of age, found that CLE-scores tended to be unchanged, paralleled by a decline of both physical activity and symptom severity [35]. These studies and our findings, indicate that a decline of self-reported respiratory symptoms in patients with EILO may be linked to a decline in the level of physical activity; i.e., not an improvement of the condition per se, but simply linked to a vanishing inducer (exercise) [24, 35, 36]. Given the relatively high prevalence of EILO, proper handling of these young individuals seems important also from a public health perspective, as it might contribute to maintenance of a healthy level of physical activity [43].

### Information and breathing advice versus inspiratory muscle training

Several authors have suggested that IBA facilitates control of EILO symptoms [7, 19], and that IMT may have an added positive influence [27]. The present study might be used to support these views, as symptoms shortly after IBA + IMT had improved in a majority, and these improvements were associated with better CLE-scores. As already discussed, this must be tested in controlled studies, as short-term contributions from IBA and IMT cannot readily be distinguished in our study design. Moreover, the improvements of self-reported symptoms after 4–6 years contribute to a positive view on both conservative treatment tools. We found no significant difference long-term between those who received only IBA and those receiving additionally 6 weeks of IMT. The IBA and IMT incorporates some of the work which in other countries might have been provided by speech and language therapists, but was provided by physicians during only one session and at rest. We were not present when participants attempted to “translate” this experience into real-life exercise at home, aspects that may have hampered long-term effects [5]. We know for example, that muscle strength is reversible and changes over time [21]. One may speculate that repeated training sessions would contribute to maintenance of the positive short-term results obtained after IBA + IMT as reported when applied in patients with paradoxical vocal fold motion at rest [45]. This should be investigated in future controlled studies.

### EILO subgroups and implications for choice of treatment

Tailored treatment based on laryngeal findings still rests on weak evidence, except in severe supraglottic EILO where laser surgery has been reported successful [20]. Subjective improvement shortly after IBA + IMT was associated with better CLE-scores, particularly at the glottic level, a finding substantiating the hypothesis that IBA + IMT mainly targets glottic obstruction [27]. Thus, it is reasonable to assume from this and previous studies that some EILO subgroups may not benefit from IBA + IMT. Indeed, a case-report suggests that IMT might even be counterproductive in severe supraglottic EILO [26]. A somewhat disappointing finding was that CLE-scores at diagnosis were not associated with self-reported symptoms 2–4 weeks after IBA + IMT, nor at follow-up 4–6 years later. Thus, diagnostic CLE-scores seemingly do not predict who will respond to IBA + IMT.

## Conclusion

Self-reported symptoms and laryngeal findings had improved 2–4 weeks after treatment with IBA plus 6 weeks of IMT. After 4–6 years, self-reported symptoms were improved compared to at time of diagnosis; however, irrespective of initial treatment and findings 2–4 weeks after IBA + IMT. Full symptom resolution was rare, and respiratory problems still disturbed most participants during physical activity. Future studies should investigate if follow-up schemes after conservative treatment, including retraining sessions with IMT, will lead to strengthened and sustained improvements.

## Supplementary Information

Below is the link to the electronic supplementary material.

Supplementary file1 (PDF 882 kb)

## Abbreviations

CI: Confidence interval
CLE test: Continuous laryngoscopy exercise test
EILO: Exercise induced laryngeal obstruction
IBA: Information and breathing advice (instructed by a physician)
IMT: Inspiratory muscle training
Pi_max_: Maximal inspiratory mouth pressure
