# Supplementary material for: Clinical responses following inspiratory muscle training in exercise-induced laryngeal obstruction
Source: Eur Arch Otorhinolaryngol. 2021 Dec 26;279(5):2511–22. doi: 10.1007/s00405-021-07214-5 (PMC8986676; doi:10.1007/s00405-021-07214-5)
Supplement: Supplementary file 1 — Supplementary file1 (PDF 882 kb) [file 405_2021_7214_MOESM1_ESM.pdf]

Online Resource

1. Title

Clinical responses following inspiratory muscle training in exercise-induced laryngeal obstruction

2. List of authors

Astrid Sandnes MD PhD[1,6], Tiina Andersen PT PhD[2, 8, 9], Hege H Clemm MD PhD[3,6], Magnus Hilland MD[4], John-Helge Heimdal MD PhD[4,5,7], Thomas Halvorsen MD PhD[3,6,9], Ola Drange Røksund PT PhD[3,4,10], Maria Vollsæter MD PhD[3,6,8]

3. Authors affiliations

[1]Department of Internal Medicine, Innlandet Hospital Trust, Gjøvik, Norway.

[2] Department of Physiotherapy, Haukeland University Hospital, Bergen, Norway

[3] Department of Pediatrics, Haukeland University Hospital, Bergen, Norway

[4] Department of Otolaryngology/Head and Neck surgery, Haukeland University Hospital, Bergen, Norway

[5] Department of Surgery, Haukeland University Hospital, Bergen, Norway

Institutes of [6]Clinical Medicine [7]Surgical Science, University of Bergen, Bergen, Norway.

[8] Thoracic Department, Norwegian Advisory Unit on Home Mechanical Ventilation, Bergen, Norway

[9] Department of Sports Medicine, Norwegian School of Sport Sciences, Oslo, Norway

[10] The Faculty of Health and Social Sciences, Western Norway University of Applied Sciences,

Bergen, Norway

4. Corresponding author:

Astrid Sandnes, Department of Medicine,

Innlandet Hospital Trust, Gjøvik, Norway

Telephone: +47 99004058, Telefax: +47 5597 5147

Email: astrid.sandnes@sykehuset-innlandet.no

Online resource 1

Inspiratory muscle training (IMT): A flow-resistive device, Respifit S®, was applied for a six weeks training period. To promote correct breathing pattern, the participants were instructed to inhale using the diaphragm and to minimize cranial shoulder movements. Two modes of IMT was applied in accordance with the manual supplied by the manufacturer , i.e. A) inspiratory muscle strength training (IMST) with the resistance set to produce mouth pressures ≥ 80% of maximal inspiratory mouth pressure (Pi_{max}) and B) moderate resistance or inspiratory muscle endurance training (IMET) with the resistance set at 60-80% of Pi_{max}. In the IMST sessions, subjects performed five maximal inhalations repeated three times, separated by a one-minute break. In the IMET sessions, subjects were instructed to breathe in and out 12-16 times for one-minute. The frequency and power were guided by an animation program ensuring correct use of the device, and each training session were stored with a memory-card for measurement of compliance. The participants trained every day, in cycles of two days with IMET followed by one day of IMST, for a total of six weeks.

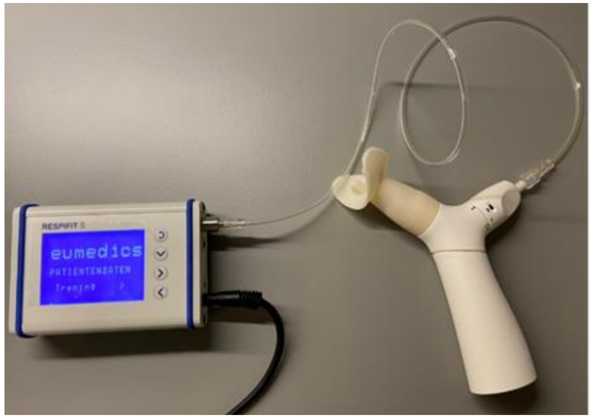

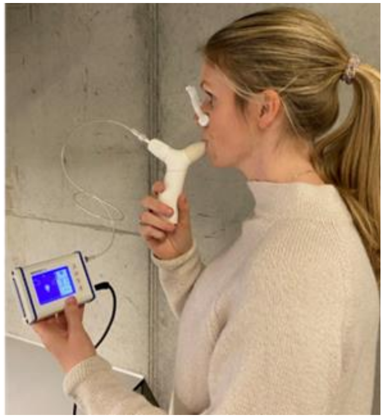

Online resource 2

QUESTIONNAIRE

FOLLOW-UP OF INDIVIDUALS WITH EXERCISE-INDUCED RESPIRATORY SYMPTOMS

Please answer all questions, and answer as to how you are feeling now.

1. Which treatment did you receive? (Tick as many boxes as appropriate)

¤ Information (about the condition) only
¤ Inspiratory muscle training
¤ Speech therapy
¤ Surgery
¤ Treatment at another clinic

2. How have your breathing problems changed since your first appointment (Q-A.1-8)?

Q-A.1 The breathing problem have got worse	□ Yes	□ No	□ Unsure
Q-A.2 Unchanged, they bother me about as much as before	□ Yes	□ No	□ Unsure
Q-A.3 Unchanged, but I have learnt to live with them	□ Yes	□ No	□ Unsure
Q-A.4 They cause me less problems because I am less active	□ Yes	□ No	□ Unsure
Q-A.5 They cause me less problems because I avoid activities that provoke symptoms	□ Yes	□ No	□ Unsure
Q-A.6 Symptoms are not as severe as before	□ Yes	□ No	□ Unsure
Q-A.7 The breathing problem has improved	□ Yes	□ No	□ Unsure
Q-A.8 I no longer have a breathing problem	□ Yes	□ No	□ Unsure

3.1 Considering all aspects of life, how much did your breathing problems effect you before you received treatment (Q-B.1)?

□ Not at all □ A little □ Quite a lot □ A great amount □ Crippling

3.2 Considering all aspects of life, how much do your breathing problems effect you now (Q-B.2)?

□ Not at all □ A little □ Quite a lot □ A great amount □ Crippling

4. How would you describe your breathing problems NOW (Q1-18)?

		**Never**	**Occasionally**	**Often**	**Nearly always**	**Always**
Q.1.	I have trouble breathing in	1	2	3	4	5
Q.2.	I have trouble breathing out	1	2	3	4	5
Q.3.	I feel tightness/pain in my throat	1	2	3	4	5
Q.4.	I feel tightness/pain in my chest	1	2	3	4	5
Q.5.	I get a hoarse voice	1	2	3	4	5
Q.6.	I frequently have a cough/clear my throat	1	2	3	4	5
Q.7.	I feel like I'm being choked	1	2	3	4	5
Q.8.	I become dizzy, nauseous and feel like I'm going to faint	1	2	3	4	5
Q.9.	The symptoms come on fast	1	2	3	4	5
Q.10.	The symptoms quickly resolve	1	2	3	4	5
Q.11.	I feel panic	1	2	3	4	5
Q.12.	I have problems breathing when I am physically active	1	2	3	4	5
Q.13.	I can hear unusual or wheezing sounds when I breathe	1	2	3	4	5
Q.14.	My symptoms prevent me from training/exercising	1	2	3	4	5
Q.15.	I become afraid when I get symptoms	1	2	3	4	5
Q.16.	My symptoms prevent me pushing myself when exercising	1	2	3	4	5

Q.17. My breathing problems continue even after I have stopped exercise/rested	1	2	3	4	5
Q.18. I can control my symptoms when I get them	1	2	3	4	5

5. How many hours per week do you play sport/ exercise at an intensity that makes you breathless and / or sweaty.

□ None
□ About 30 minutes
□ About 1 hour
□ About 2-3 hours
□ About 4-6 hours
□ 7 hours or more

6.1 What sport do you play?____

6.2 At what level?

□ For personal training
□ Local
□ Regional
□ National
□ International

7. Have you experienced any side effects/ negative symptoms after treatment for your exercise induced breathing problems?

□ no prolonged side effects / problems
□ Yes
If yes, please describe:

Thank you for taking the time to answer!

Online resource 3

Table 5: **Demographic data** obtained from eligible subjects with exercise induced laryngeal obstruction (EILO) diagnosed at the outpatient clinic at Haukeland University Hospital, Bergen, Norway. Data are from time of diagnosis and divided in those who answered a follow-up questionnaire 4-6 years after diagnosis ("participants") and those who did not answer the follow-up questionnaire ("Non-responders").

	Participants			Non-responders			
	**IBA**	**IBA+IMT**	**P-value[A)]**	**IBA**	**IBA+IMT**	**P-value[B)]**	**P-value[C)]**
Number	23	32		25	18		
Male/Female	7/16	3/29		11/14	2/16		
Age at diagnosis, mean (range)	15.2 (12-21)	17.5 (10-30)	0.019*	16.0 (12-27)	16.6 (11-23)	0.248	0.269
Age at follow-up, mean (range)	20.4 (17-26)	22.7 (15-36)	0.036*	21.4 (18-33)	21.7 (17-28)		
Age symptom debut, mean (range)	10.5 (5-15)	12.6 (1.5-20)	0.052	13.1 (3-26)	12.7 (12-14)	0.097	0.855
Activity hour(s) per week, median	≥7 hours	≥7 hours	0.667[D)]	4-6 hours	≥7 hours	0.181 [D)]	0.720 [D)]
Activity times per week, median	4-6 x/week	4-6x/week	0.496[D)]	4-6 x/week	4-6 x/week	0.064 [D)]	0.683 [D)]
Level of sports activity, at diagnosis†							
No organized activity	3	2		6	2		
Local/regional level	16	16		11	6		
National level	3	10		2	8		
International level	0	4		0	2		
[E)]Effected by breathing problem, mean (95% CI)							
When physically active	3.6 (3.2-4.0)	3.6 (3.3-3.9)	0.562	3.2 (2.7-3.7)	3.5 (2.9-4.1)	0.599	0.768
Considering all aspects of life	2.9 (2.4-3.5)	2.9 (2.5-3.2)	0.793	2.5 (2.1-3.0)	2.5 (1.4-3.6)	0.688	0.932
CLE-score (total) at diagnosis, mean (95% CI)	2.7 (2.3-3.2)	3.8 (3.4-4.2)	0.007*	2.1 (1.5-2.7)	3.1 (2.5-3.8)	0.196	0.227

IMT: inspiratory muscle training, IBA: information and breathing advice, CI: confidence interval, CLE score: grading of laryngeal obstruction according to Maat et al [1],

A) Comparison between responders IBA-group and IBA+IMT group using student t-test and presented as p-value. B) Comparison between responders and non-responders in IBA and C) IBA+IMT group using Student's t-test. D) Comparison with non-parametric Mann-Whitney-U-test as the data was non-normally distributed

E) "How much do your breathing problem effect you?" at time of diagnosis, answer scale 1-5: (1: never, 2: occasionally, 3: often, 4: nearly always, 5: always).

† Items where numbers do not add up to the total group n are due to missing answers on questionnaire.

Reference

1. Maat, R.C., et al., *Audiovisual assessment of exercise-induced laryngeal obstruction: reliability and validity of observations.* Eur Arch Otorhinolaryngol, 2009. **266**(12): p. 1929-36.
2. Heimdal, J.H., et al., *Continuous laryngoscopy exercise test: a method for visualizing laryngeal dysfunction during exercise.* Laryngoscope, 2006. **116**(1): p. 52-7.
